# Cytomegalovirus Reactivation Is Associated With Lower Rates of Hepatocellular Carcinoma Recurrence After Liver Transplantation

**DOI:** 10.3389/ti.2025.14553

**Published:** 2025-06-10

**Authors:** Victoria Aguilera, Sarai Romero Moreno, Isabel Conde, Angel Rubín, Angela Carvalho-Gomes, Mario Romero, Javier Zamora-Olaya, Miguel Angel Gómez-Bravo, Esteban Fuentes-Valenzuela, Cristina Dopazo, Nikita Bilbao, Antonio González, Ana Sánchez-Martínez, Sonia Pascual, Jesús Rivera-Esteban, José Ignacio Herrero, Sara Lorente, Antonio Cuadrado-Lavín, Flor Nogueras, Laura Martínez-Arenas, Rocío González-Grande, Marina Berenguer, Manuel Rodriguez-Perálvarez

**Affiliations:** ^1^ Hepatology and Liver Transplant Unit, La Fe Universitary and Politécnic Hospital, Instituto de Investigación Sanitaria (IIS) La Fe, Centro de Investigación Biomédica en Red de Enfermedades Hepáticas y Digestivas (CIBEREHD), Faculty of Medicine, Valencia University, Valencia, Spain; ^2^ Hepatology and Liver Transplant Unit, La Fe Universitary and Politécnic Hospital, Valencia, Spain; ^3^ Hepatology and Liver Transplant Unit Trasplante Hepático, La Fe Universitary and Politécnic Hospital, Instituto de Investigación Sanitaria (IIS) La Fe, Centro de Investigación Biomédica en Red de Enfermedades Hepáticas y Digestivas (CIBEREHD), Valencia, Spain; ^4^ Laboratorio de Hepatología, Cirugía HBP y Trasplantes, Instituto de Investigación Sanitaria (IIS) La Fe, Centro de Investigación Biomédica en Red de Enfermedades Hepáticas y Digestivas (CIBEREHD), Valencia, Spain; ^5^ Department of Hepatology and Liver Transplantation, HUGregorio Marañón, Centro de Investigación Biomédica en Red de Enfermedades Hepáticas y Digestivas (CIBEREHD), Madrid, Spain; ^6^ Department of Hepatology and Liver Transplantation, Hospital Universitario Reina Sofía, Instituto Maimonides de Investigación Biomédica de Córdoba (IMIBIC) and University of Córdoba, Córdoba, Spain; ^7^ Hospital Virgen del Rocio, Unidad de Cirugía Hepatobilipancreática, Sevilla, Spain; ^8^ Unidad de Hepatología y Trasplante Hepático, HURio Hortega, Valladolid, Spain; ^9^ Department of HPB Surgery and Transplants, Vall d’Hebron Hospital Universitari, Vall d’Hebron Institut de Recerca (VHIR), Vall d’Hebron Barcelona Hospital Campus, Universitat Autónoma de Barcelona, Barcelona, Spain; ^10^ Department of Hepatology, Hospital Universitario Ntra. Sra. de la Candelaria, Tenerife, Spain; ^11^ Liver Transplantation Unit, Hospital Universitario Virgen de la Arrixaca and IMIB, Murcia, Spain; ^12^ Unidad de Hepatología y Trasplante Hepático, Instituto de Investigación Sanitaria y Biomédica de Alicate (ISABIAL), Centro de Investigación Biomédica en Red de Enfermedades Hepáticas y Digestivas (CIBEREHD), HGU Dr Balmes, Alicante, Spain; ^13^ Department of Gastroenterology and Hepatology, Hospital Universitario Puerta de Hierro, Instituto de Investigación Sanitaria Puerta de Hierro ‐ Segovia de Arada (IDIPHISA), Majadahonda, Spain; ^14^ Liver Unit, Clínica Universidad de Navarra and IdiSNA, Centro de Investigación Biomédica en Red de Enfermedades Hepáticas y Digestivas (CIBEREHD), Pamplona, Spain; ^15^ Department of Hepatology and Liver Transplantation, Hospital Clínico Lozano Blesa, University of Zaragoza and ISS Aragón, Zaragoza, Spain; ^16^ Gastroenterology and Hepatology Department, Clinical and Translational Research in Digestive Diseases, Valdecilla Research Institute (IDIVAL), Marqués de Valdecilla University Hospital, Cantabria University, Santander, Spain; ^17^ Department of Hepatology and Liver Transplantation, Hospital Virgen de las Nieves, Granada, Spain; ^18^ Hepatology, Hepatobiliopancreatic Surgery and Transplant Laboratory, Instituto de Investigación Sanitaria (IIS) La Fe Health Research Institute, Universitat Politècnica de València, Centro de Investigación Biomédica en Red de Enfermedades Hepáticas y Digestivas (CIBEREHD), ISCIII, Valencia, Spain; ^19^ Department of Hepatology and Liver Transplantation, Hospital Regional Universitario de Málaga, Málaga, Spain; ^20^ Department of Hepatology and Liver Transplantation, Hospital Universitario Reina Sofía, University of Córdoba, Instituto Maimonides de Investigación Biomédica de Córdoba (IMIBIC), Centro de Investigación Biomédica en Red de Enfermedades Hepáticas y Digestivas (CIBEREHD), Córdoba, Spain

**Keywords:** liver transplantation, cytomegalovirus, survival, hepatocellular carcinoma, donation after circulatory determination of death, Immunosupression

## Abstract

In patients with hepatocellular carcinoma (HCC), undergoing liver transplantation (LT), cytomegalovirus reactivation (CMVr) may modulate the immune system to prevent tumor recurrence. In this multicenter retrospective study (2010–2015) involving 15 institutions, we assessed the effect of early CMVr in tumor recurrence rates among 771-LT HCC patients with tacrolimus-based immunosuppression (88% men, mean age 58 years). CMV prophylaxis was implemented for 19.7% of patients, while the rest were managed with preemptive therapy. The Milan criteria were met by 88% of patients. Microvascular invasion was present in 12.7% of explanted livers. The serum AFP level before transplantation was 5.1 (3–15) ng/mL. After a median follow-up of 7.4 years, 101 patients (13%) experienced HCC recurrence. CMVr occurred in 235 patients (30.5%) at a median of 41.5 days post-LT and 42 patients (5.6%) had CMV disease. Cumulative exposure to tacrolimus within the first 3 months after LT was similar among patients with and without CMVr. In a multivariate Cox regression analysis, factors associated with an increased rate of HCC recurrence included microvascular invasion [HR:2.82, CI95%:1.55–5.14; p 0.0001], donation after circulatory determination of death [HR:4.43,CI95%:1.52–12.9; p 0.006) and diameter of the main nodule at explant [HR:1.04, CI95%:1.02–1.06; p < 0.001]. Meanwhile CMVr [HR:0.46, CI95%:0.23–0.93, p 0.031] and MELD [HR:0.93, CI95%:0.87–0.99; p0.017] exhibited protective effects. In conclusion, early CMVr may protect against HCC recurrence. The underlying immune mechanisms warrant further investigation.

## Graphical Abstract

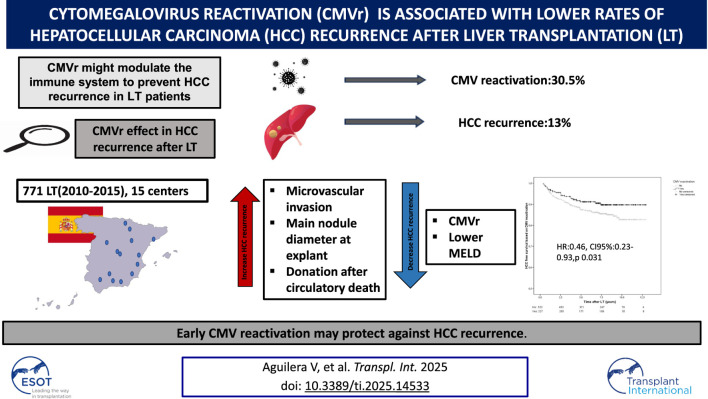

## Introduction

Recurrence of hepatocellular carcinoma (HCC) following liver transplantation (LT) occurs in approximately 8%–20% of Well‐selected patients is an accepted terminology in HCC and LT in literature [1–4]. Clinical, pathological, and biological factors influence the risk of HCC recurrence [3] with several imperfect models proposed to assess this risk both pre- and post-LT [1, 2, 5, 6]. In addition, there are no established surveillance guidelines for HCC recurrence and there is a significant heterogeneity across different institutions [6, 7]. Transplanting within the MILAN criteria mitigates the risk; however, the majority of centers are now expanding the criteria. Because exposure to immunosuppressive drugs, particularly calcineurin inhibitors (CNIs) early after LT, is also associated with oncogenesis in a dose-dependent manner via impairment of the immune surveillance [8–10], tacrolimus minimization and addition of mammalian target of rapamycin inhibitors (mTORi) is a strategy used by some centers albeit with limited benefit [8, 9, 11].

Established factors favoring HCC recurrence include microvascular invasion and high alpha-fetoprotein (AFP) levels, along with tumor numbers and size. Although cytomegalovirus (CMV) infection is the most common opportunistic infection in LT recipients and remains a cause of life-threatening disease and allograft rejection [12, 13], recent studies have suggested a potential beneficial effect of CMV reactivation (CMVr) in some tumors [14, 15]. Immune modulation and modification of the tumor microenvironment following CMVr could be responsible for tumor control in this scenario [16–20].

In the present retrospective multicenter cohort study, we aimed to evaluate whether the occurrence of CMVr after LT in patients with HCC has a potential effect against tumor recurrence.

## Materials and Methods

### Study Subjects and Analyzed Variables

This is a retrospective multicenter study that involved 15 institutions representing 62% of Spanish LT activity. Patients undergoing LT due to HCC between January 2010 and December 2015 under tacrolimus-based immunosuppression, were consecutively included. The MILAN criteria were used by the majority of centers during the study period [21]. Exclusion criteria were age <18, re-transplantation, combined organ transplantation, death within the first 6 months after LT, and relevant missing data concerning CMVr or HCC recurrence. Patients were followed until death or November 2022, whichever occurred first.

HCC was confirmed by the pathological examination of the explanted liver in all cases with the exception of patients showing complete tumor necrosis related to pre-LT locoregional bridging therapies, with previous radiological HCC diagnosis according to international guidelines.

The study protocol was approved by the Ethics Committee of Clinical Research of La Fe Universitari and Politécnic Hospital (ref number: 2022-601-1) and was conducted in accordance with the 1975 Helsinki Declaration. Given the retrospective nature of the data, the ethics committee waived the need for informed consent at the other participating hospitals.

Collected variables included donor and recipient serology, donor and recipient mismatch, pre-emptive therapy, CMVr after LT, primary infection, CMV disease and the need for antiviral therapy.

The main risk variable was CMVr, which was defined as a detectable viral DNA above the local quantification threshold after LT. We also recorded CMV primary infection for descriptive purposes, which was defined as a positive post-LT viral CMV DNA in a patient with a negative CMV serology test before LT. To study the relationship between CMV and HCC recurrence, we considered CMVr a more appropriate risk factor because it is an accepted surrogate for lower immune system awareness; in contrast, CMV primary infection, which is primarily related to Donor-Recipient mismatch, was controlled as a potential confounder in the multivariable analysis.

The main study outcome was HCC recurrence as a time-dependent event accounting for the interval between LT and imaging or pathological diagnosis of tumor recurrence, whichever occurred first. The secondary outcomes were disease-free survival and overall survival rates.

Other collected variables associated with HCC recurrence and death included:

(i) Related to the donor: demographics and type of donor.
(ii) Related to the recipient: sex, age, indication for LT, functional MELD score, presence of renal failure (estimated glomerular filtration rate [eGFR] < 60 mL/min), cardiovascular risk factors (arterial hypertension, diabetes mellitus), and human immunodeficiency virus (HIV) infection.
(iii) Related to HCC: bridging and/or downstaging to the Milan criteria, type of locoregional therapy (radiofrequency ablation, chemoembolization, radioembolization, combination therapy), pathological features at explant including microvascular invasion and grade of differentiation, AFP levels at listing and at LT, number of nodules, and diameter of the largest nodule in both the radiological assessment and the explanted liver.
(iv) Immunosuppression: in a subgroup of patients who participated in a previous study [8, 10], cumulative exposure to tacrolimus, defined as the area under curve of trough concentrations within the first 3 and 12 months after LT was obtained.
(v) Patient and graft survival and cause of death.

### Variable Definitions

CMVr or primary infection were defined as detectable viral DNA above the local quantification threshold after LT. We defined primary infection as occurring in those patients witha negative CMV serology test before LT.

CMV disease was defined using internationally agreed-upon criteria, including the presence of appropriate clinical symptoms and documentation of CMV in tissue using different techniques (histopathology, virus isolation, immunohistochemistry, or nucleic acid hybridization) [22].

We collected the first positive CMV viral load (VL) and the peak VL (defined as the highest detectable DNAemia per each patient) in both CMVr and primary infection. We also collected the median CMV VL in those patients who were treated with antivirals.

### Management of CMVr and HCC Surveillance

Prophylaxis with valganciclovir (900 mg once daily) was administered within the first 3–6 months after LT to CMV-negative patients who had received a CMV-positive donor liver. All remaining patients underwent only CMV DNA surveillance. Serial blood samples were obtained weekly during the first month, every 2 weeks from months 1–3, and at the time of clinical visits thereafter. CMV surveillance lasted for the first 6–12 months. Preemptive therapy with valganciclovir (900 mg bd) was implemented immediately after patients showed detectable and/or an increasing CMV viremia without a prespecified threshold or when an upward trend was observed for both primary infection or reactivation and maintained up to the confirmation of two consecutive negative samples, at least 4 weeks apart [12].

Surveillance of HCC recurrence after LT was performed according to each center’s practice by combining serum AFP and imaging techniques. The majority of centers used abdominal ultrasounds and/or whole-body computed tomography scans performed at least every 6 months for the first 2–5 years after LT depending on risk factors.

All patients received tacrolimus-based immunosuppression and tapering corticosteroids, which were withdrawn between the third and sixth months after liver transplantation, except in cases of autoimmune disease, where the lowest tolerated dose was maintained. The majority of centers did not implement specific protocols for patients with HCC. Seven centers used everolimus as part of the immunosuppression protocol, which was introduced in week 4 post-LT [11] in patients with poor prognostic factors.

### Statistical Analysis

Continuous variables were summarized as mean and standard deviation (SD) or median and interquartile range (IQR) as appropriate. Categorical variables were presented as absolute numbers and frequencies. Normal distribution of variables was assessed using the Kolmogorov-Smirnov test. A Student’s t-test was used for quantitative variables, and a Chi-square and Fisher’s exact test were used for categorical variables.

Patient survival analysis was performed with Kaplan-Meier survival curves.

The initial multivariable model included the variables with p-values <0.10 in the univariate analysis. Variables with a p-value above this threshold could be included if they were considered clinically relevant by the investigators or if found to be related to HCC recurrence in previous studies. Regarding HCC morphological variables, we included those available at baseline after the analysis of the explanted liver (number of nodules, diameter of the main nodule) excluding models that combined some of these morphological variables.

Patients with AFP >1000 ng/mL (n = 6) were excluded from the regression analysis to avoid distortion and inconsistencies due to edged values.

The significance level was set at 5% (p < 0.05) for all analyses.

Data analysis was performed using SPSS version 22.0 (IBM, Chicago, USA).

## Results

**FIGURE 1:**
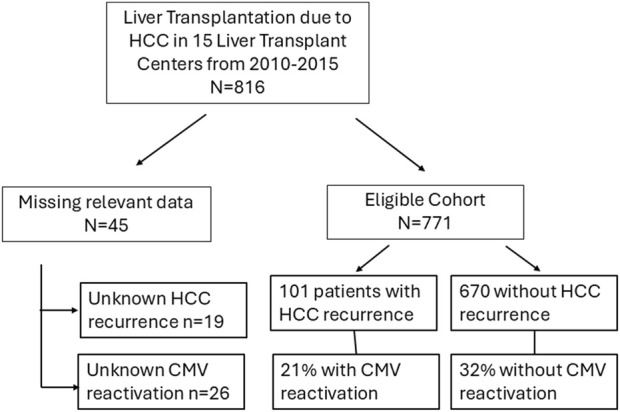
Flow chart of the study population.

The eligible cohort comprised a total of 771 LT patients with HCC on explant out of an initial cohort of 816 patients, from 15 Spanish institutions. Forty-five patients with missing data relevant to the analysis [CMVr (n = 19) or HCC recurrence (n = 26)] were excluded from the analysis. The median follow-up was 7.4 years (IQR 4.9–9.1) after LT. The flowchart showing the study population is represented in Figure 1.

Baseline features of the included cohort are shown in Table 1. The majority of patients were men (n = 681, 88%), with a median age of 58.7 years (IQR 53.8–63.6) at LT. The median donor age was 62 (IQR 49–73) years. The majority of patients received a brain-dead donor liver (n = 745, 97.5%). The most frequent etiologies of liver disease that led to LT were alcohol and hepatitis C virus (HCV) (n = 399, 52% and n = 390, 50.5%, respectively). The median MELD score at LT was 12 (IQR 9–16). More than a third of patients were diabetic at the time of LT and the median eGFR was 91.2 mL/min (IQR 80–100).

**TABLE 1:** Pre-transplant features.

Baseline features (n = 771)	
Age (years), median (IQR)	58.7 (53.8–63.6)
Sex (% men)	681 (88%)
Donor age (years), median (IQR)	62 (49–73)
Type of donor, n (% of Brain death)	745 (97.5%)
Etiology of cirrhosis, n (%) HCV Alcohol HBV MASH	390 (50.5%) 399 (52%) 65 (8.4%) 19 (2.5%)
MELD score at LT (median, IQR)	12 (9–16)
Diabetes mellitus (with oral antidiabetics or insulin), n (%)	274 (36%)
eGFR ml/min, median (IQR)	91 (80–100)
Duration of follow up (years), median (IQR)	7.4 (4.9–9.1)

HCV, Hepatitis C virus; HBV, Hepatitis B virus; eGFR: estimated-glomerular filtrate rate; LT, liver transplantation; MASH, metabolic dysfunction-associated steatohepatitis.

Regarding HCC features before LT and at the time of explant (Table 2), the majority of patients met the Milan criteria (88%) or the Up-to-Seven criteria (98%). The median AFP at inclusion was 6 ng/mL (IQR 3.4–17). A high proportion of patients were treated with locoregional therapy, with transarterial chemoembolization and radiofrequency ablation being the most common (36.2% and 18%, respectively). A minority of patients (13.4%) was waitlisted after downstaging. Microvascular invasion was present in 12.7% of the explants and more than half of the HCCs were moderately or poorly differentiated (48% and 7%, respectively).

**TABLE 2:** Hepatocellular (HCC) features in the overall cohort (n = 771).

Bridging, n (%) Transarterial chemoembolization Radiofrequency ablation Radioembolization Combination therapy None	279 (36.2%) 139 (18%) 7 (1%) 8 (8%) 252 (33%)
Downstaging, n (%)	103 (13.4%)
AFP at WL inclusion (ng/mL)(median, IQR)	6 (3.4–17)
AFP at LT (ng/mL) (median, IQR)	5.1 (3–15)
Milan “in” Criteria (n = 753), %	664 (88%)
Up to Seven Criteria (n = 755), %	737 (98%)
Retreat Score (n = 549) 0-3 points 4-8 points	443 (81%) 106 (19%)
Number of nodules at imaging (median, IQR) Size of larger nodule at imaging(mm), (median,IQR) Number of viable nodules at pathology (mm), (median, IQR) Size of the largest nodule at pathology (mm)	1 (1–2) 22 (15–30) 1 (1–2) 20 (12–18)
Microscopic intravascular invasion at pathology, n (%)	98 (12.7%)
Differentiation grade, n (%) Well differentiated Moderate differentiation Poor differentiated Complete necrosis	218 (30%) 343 (48%) 49 (7%) 108 (15%)

AFP, alpha-fetoprotein; WL, waiting list.

CMV-related features are shown in Table 3. Both CMV serologies of donors and recipients were positive in 67.5% of patients. A donor-recipient mismatch (D+/R−) was found in 11.5% of patients, negative donor with positive recipient in 18.6% of patients and negative donor with negative recipient in 2.4% of patients. Approximately one-third of patients (30.9%) had CMVr at a median of 41.5 days (IQR 26–56) but only 5.6% of these patients developed CMV disease. Antiviral therapy against CMV was administered to 66% of those with reactivation or primary infection and the remaining patients were managed with reduction of immunosuppression only or exhibited spontaneous clearance. The first detectable and the peak VL were higher in those with primary infection as opposed to patients with CMVr, regardless of subsequent antiviral therapy (see Table 3).

**TABLE 3:** Cytomegalovirus related features (n = 771).

Mismatch CMV, n (%) D/R +/+ D/R +/− D/R−/+ D/R−/−	421 (67.5%) 72 (11.5%) 116 (18.6%) 15 (2.4%)
CMV Prophylaxis n (%)	148 (19.7%)
CMV primary infection, n (%)	47 (6.1%)
CMV reactivation (CMVr), n (%)	235 (30.5%)
First positive CMV VL (median, IQR) (UI/mL) In patients with CMVr In patients with primary infection	758 (405–2,340) 4,849 (1,590–25800)
Peak CMV VL (UI/mL) (median, IQR) In patients with CMVr In patients with primary infection)	1915 (604–6,941) 7,084 (3,265–34214)
First positive CMV VL (median, IQR) (UI/mL) if followed by antiviral therapy In patients with CMVr In patients with primary infection	906 (408–3,310) 4,035 (1,362–34214)
Peak CMV VL (median, IQR) (UI/mL) if followed by antiviral therapy In patients with CMVr In patients with primary infection	2,978 (1,026–11000) 8,938 (2,810–49600)
Time to CMV reactivation (days, median, IQR)	41.5 (26–56)
CMV disease, n (%)	42 (5.6%)
Need of antiviral treatment, n (%) In those with primary infection or reactivation	187 (66.3%)

CMV: cytomegalovirus, CMVr: CMV, reactivation; D: donor, R: recipient, VL: viral load.

HCC recurrence occurred in 13.1% (n = 101) of patients after a mean of 2.78 (SD +/−2.3) years. HCC recurrence-free survival at 1, 3, 5, and 7 years after LT was 96%, 91.6%, 88.2% and 86.8%, respectively (Supplementary Figure S2). The overall survival rate for patients without HCC recurrence was 99.5%, 92.1%, 86.7% and 81.5% at 1, 3, 5, and 7 years, respectively, which was significantly higher than that for patients with HCC recurrence (96,9%,62.9%,38.1% and 21.6% at 1, 3, 5, and 7 years, respectively) (Log Rank p < 0.05) (Supplementary Figure S3).

Data on immunosuppression, cumulative exposure to tacrolimus and rejection were available for 324 patients (42% of the entire cohort). We decided not to perform a sensitivity analysis in this sub-cohort because it showed different HCC features, and the number of HCC recurrence events was insufficient to allow meaningful comparisons. Of note, patients with and without CMV reactivation showed comparable cumulative exposure to tacrolimus within the first 3 months after LT, with 41% and 43% of patients, respectively, stratified as receiving high tacrolimus exposure (p = 0.497). In contrast, a high cumulative exposure to tacrolimus was associated with increased HCC recurrence rates. Basiliximab was used by 13.6% of subjects in the studied cohort and overall, 11% of patients were treated with mTORi. In total 16% of patients developed a biopsy-proven acute cellular rejectionduring study period.

The predictors of post-LT HCC recurrence in the univariate and multivariable Cox-regression analyses (performed on 465 patients with available data on all the variables included) are shown in Supplementary Table S1 and in Table 4, respectively. Of note, we reproduce the multivariable analysis after excluding variables with missing values in more than 10% of patients, namely (AFP at WL, AFP at LT, Number of nodules at explant, and cumulative exposure to tacrolimus at months 3 and 12). The new analysis included 615 patients (79.8% of the entire study population) and produced consistent results regarding the protective effect of CMVr against HCC recurrence (HR = 0.57; p = 0.037) and recurrence-free survival (HR = 0.77; p = 0.112) (data not shown).

**TABLE 4:** Cox Regression model for variables associated with HCC recurrence.

Variable	HR	95% IC	p-value
Sex Men Women	1 1.26	0.44–3.57	0.671
Recipient age (years)	0.97	0.93–1.01	0.088
Donor type Circulatory death	4.43	1.52–12.9	0.006**
HBV etiology	0.84	0.34–2.11	0.717
MELD score	0.93	0.87–0.99	0.017*
AFP at WL	1	1–1	0.961
AFP at LT	1	1–1	0.721
Nodule size at last imaging before LT	1.03	0.77–1.37	0.843
Number of nodules at last imaging before LT	0.99	0.97–1.01	0.264
Nodule size at explant	1.04	1.02–1.06	<0.001***
Number of nodules at explant	1.03	0.97–1.10	0.259
CMV reactivation	0.46	0.23–0.93	0.031*
Micro-vascular invasion at explant	2.82	1.55–5.14	0.001**
Differentiation grade Moderate or poor	1.41	0.79–2.52	0.248

*p < 0.05; **p < 0.01; ***p < 0.001, The multivariate final model was made with 465 LT patients.

CMV, cytomegalovirus; HBV, Hepatitis B virus; LT; liver transplantation; WL, waiting list.

**FIGURE 2:**
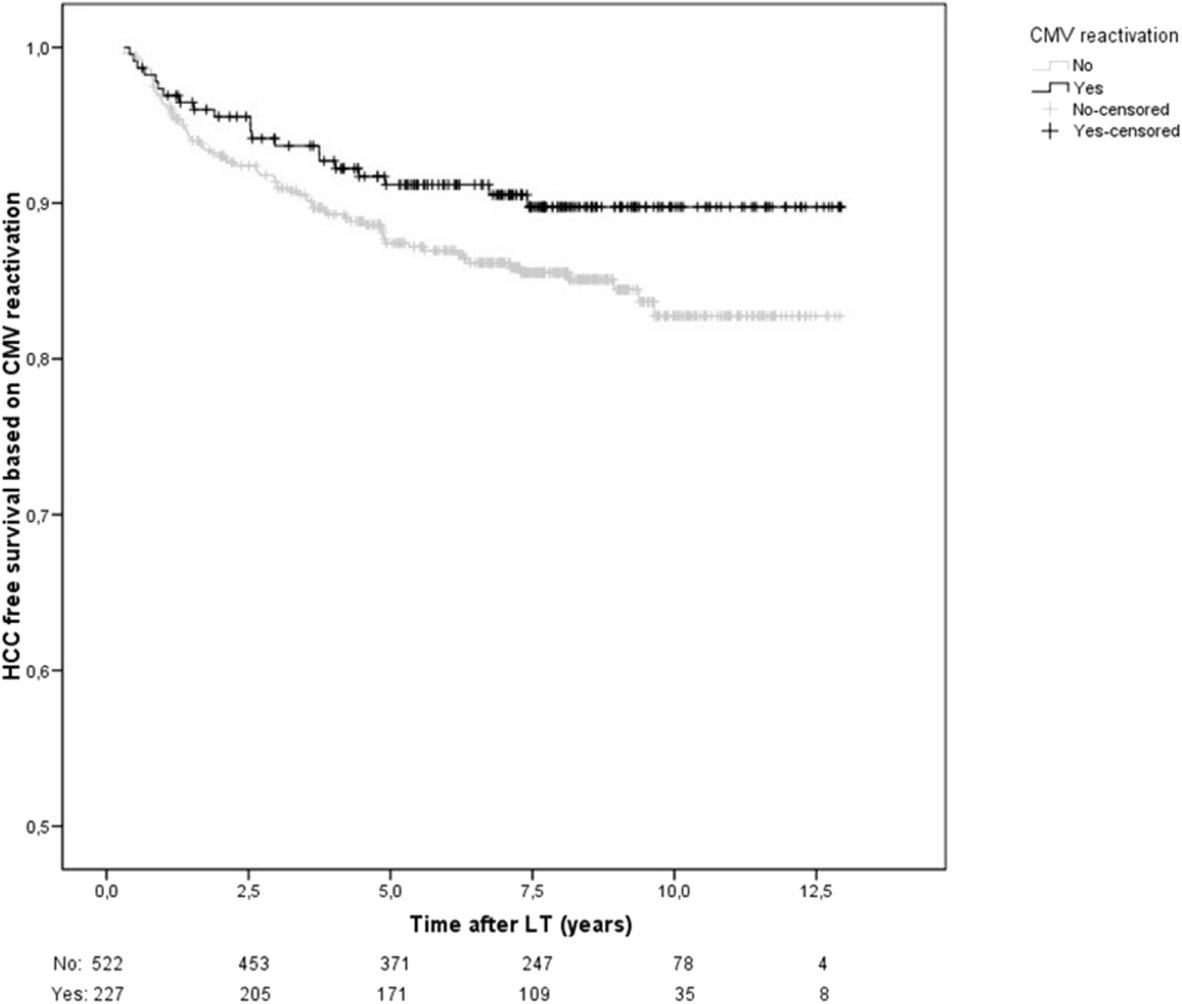
HCC-recurrence free survival based on CMV reactivation. HCC recurrence free survival was significantly higher in those with CMVr (HR 0.46, 95%CI 0.23–0.93, p = 0.031).

Factors independently associated with an increased risk of HCC recurrence were donation after circulatory determination of death (HR 4.43, 95%CI 1.52–12.9, p = 0.006), diameter of the main nodule at explant (HR 1.04, 95%CI 1.02–1.06, p < 0.001) and microvascular invasion (HR 2.82, 95%CI1.55-5.14, p = 0.001) while lower MELD scores at transplant (HR 0.93; 95%CI 0.87–0.99, p = 0.017), and CMVr (HR 0.46, 95%CI 0.23–0.93, p = 0.031) having a protective effect. Patients with CMVr had better HCC free-survival than those without CMVr after LT (Figure 2). However, CMV primary infection was not associated with lower HCC recurrence.

In addition, we also explored whether CMV mismatch or the need for antiviral treatment after CMVr (antiviral treatment vs. spontaneous clearance) could have affected HCC recurrence. For CMV mismatch, an additional exploratory analysis was conducted in which no association was found (HR: 1.69, 95%CI 0.8–3.57, p = 0.169). An alternative multivariable model was built to test the interaction between CMVr and antiviral treatment, which did not obtain statistical significance (p = 0.534), meaning that the decision to treat or not CMVr may not have an influence on HCC recurrence rates. In addition, we stratified our study population according to the occurrence of CMVr and antiviral therapy usage into three groups: patients without reactivation, patients with untreated reactivation, and patients with treated reactivation. The multivariable Cox model showed no statistically significant difference in the risk of HCC recurrence between patients with treated (HR = 0.28, p = 0.22) or untreated CMVr (HR = 0.49, p = 0.07), and the reference group (non-reactivated) (Supplementary Table S3).

**FIGURE 3:**
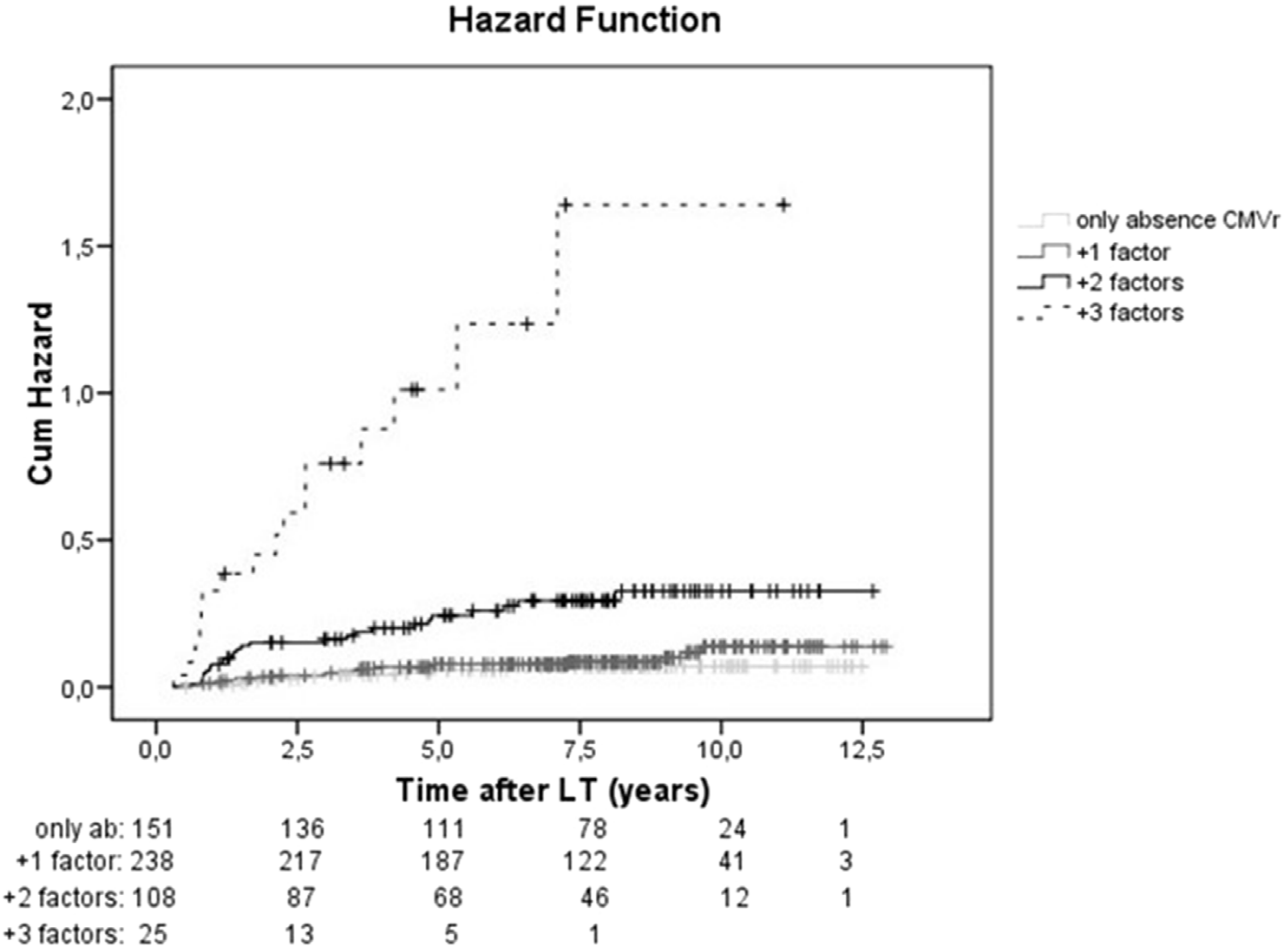
Cumulative risk of HCC recurrence stratified according to risk factors. Kaplan-Meier curve showing the cumulative risk of hepatocellular carcinoma recurrence after liver transplantation. For the analysis, the study population was stratified according to the number of risk factors, which included: absence of reactivation of cytomegalovirus (CMVr), size of the main nodule at explant, MELD score and microvascular invasion. (light grey solid line: only absence of CMVr, dark grey solid line: absence of CMVr and 1 added factor, black solid line: absence of CMVr and 2 added factors, absence of CMVr and 3 added factors: black dashed line).

A Kaplan-Meier curve with the cumulative risk of hepatocellular carcinoma recurrence after liver transplantation is shown in Figure 3. The study population was stratified by the number of risk factors, which included absence of CMV reactivation, size of the main nodule at explant, higher MELD score and microvascular invasion. The sum of the predictive clinical factors had an incremental effect on the risk of HCC recurrence (HR = 3.07, p < 0.001) (Figure 3). Adding one factor to the absence of CMVr had a modest effect on HCC recurrence rate (HR = 1.4, p = 0.361), but the addition of 2 or 3 factors resulted in a significant increase in the risk of HCC recurrence (HR 4.51, p < 0.001 and HR 21.5, p < 0.001, respectively).

A total of 237 patients (30.7%) died, and the main cause of death was HCC recurrence (n = 84), 41%) followed by *de novo* tumors (n = 50, 24%) (Table 5). Variables associated with survival in the univariate analysis were donor age, recipient age, sex, alcohol etiology, Milan criteria, Retreat score, AFP levels at listing and at LT, tumor burden, microvascular invasion, tumor differentiation grade and CMVr (Supplementary Table S2). In the multivariable analysis, increased AFP at LT, and diameter of the main nodule at explant were associated with reduced survival, while CMVr reduced the risk of death by 33%, (HR 0.67, P = 0.049) (Table 6).

**TABLE 5:** Outcomes after LT.

HCC recurrence (%, IC95%)	101 (13.1%, 10–15.5)
Time to recurrence (years, SD)	2.78, 2.3
Death (%)	237 (30.7%)
Causes of Death (%) Disease recurrence (n,%) De *novo* tumors (n,%) CVE (n,%) Others (n,%)	84 (40.8%) 50 (23.8%) 10 (4.9%) 64 (30.8%)

HCC, hepatocelullar carcinoma; CVE, cardiovascular events.

**TABLE 6:** Cox regression model of factors associated with survival.

Variable	HR	95% CI	p-value
Donor age (years)	1.00	0.99–1.02	0.429
Recipient age (years)	1.01	0.98–1.03	0.676
Sex (men)	1.11	0.58–2.13	0.758
HCV etiology	0.83	0.55–1.25	0.378
Alcohol etiology	1.12	0.75–1.68	0.571
AFP at WL	0.998	0.995–1.002	0.295
AFP at LT	1.002	1.001–1.004	0.009**
Number of nodules (preLT imaging)	1.03	0.86–1.24	0.755
Nodule size at explant	1.018	1.01–1.03	0.001**
Number of nodules at explant	0.98	0.91–1.05	0.577
Micro-vascular invasion	1.42	0.91–2.12	0.120
Differentiation grade Moderate or poor	1.26	0.89–1.79	0.188
CMV reactivation	0.67	0.45–0.99	0.049*

*p < 0.05; **p < 0.01; ***p < 0.001, The multivariate final model was based on 481 LT, patients.

CMV, cytomegalovirus; HCV, Hepatitis C virus; HBV, Hepatitis B virus; LT, liver transplantation; WL, waiting list.

## Discussion

Although HCC recurrence accounts for a small percentage of patients, it significantly impacts survival. Identification of specific factors before and/or after LT that can be modified to enhance prognosis is an active area of research [9–11]. This multicenter retrospective observational Spanish study, involving a large number of patients, reveals that CMV reactivation is associated with a lower rate of HCC recurrence after LT. Other well-described factors such as microvascular invasion and nodule size at explant, were also significantly associated with recurrence in our study [23, 24]. Of note, treatment of CMVr did not influence HCC recurrence. In addition, CMVr was also associated with improved overall survival further strengthening the association.

Pathophysiological explanations for the role of CMV in modulating the tumor microenvironment have been hypothesized. A potential oncolytic effect of CMV inducing remission, ablation, or tumor death has been postulated through different mechanisms such as stimulating cytokine inhibition, interfering with tumor extravasation, or tumor vascularization taking a multimodal approach. In mouse models of melanoma and HCC, CMV infection showed clearance of the established tumor [18, 25, 26]. Specifically, in a murine model of HCC cells (HepG2), Kumar et al. demonstrated that CMV infection of the HCC cells resulted in the absence of tumor or limited tumor growth by promoting cancer cell apoptosis through the activation of caspases [26]. Other studies have shown that CMV reactivation induces tumor cell apoptosis directly or by stimulating cytokines and antitumor immune responses [19]. Cross-reactivity between CMV-stimulated innate and adaptive immune responses and cancer cells has also been reported. Natural killer cells and Vδ2 ^neg^ϒδ T cells have been reported to expand when stimulated by CMV reactivation, with the subsequent ability to kill both CMV-infected cells and carcinoma cells *in vitro* due to the shared reactivity of the Vδ2 ^neg^ϒδ T cells against CMV-infected cells and tumor intestinal epithelial cells [16, 27]. Additionally, the role of CMV-specific CD8 T cell responses in targeting tumors with CMV epitope-conjugated viral antigens presented by HLA-I has been described [28].

In oncological clinical scenarios, the protective effect of CMV reactivation has also been described. Takenaka et al. showed a beneficial effect in allogeneic hematopoietic stem cell transplantation for acute myeloid leukemia. CMV reactivation decreased the risk of relapse (20% vs. 26.4%, p: 0.027). This anti-leukemic effect was attributed to the CMV-driven expansion of donor-derived memory-like NKG2C + NK and Vδ2 negϒ δT cells, which demonstrated an ability to kill both infected CMV cells and leukemic cells due to shared reactivity [29]. This effect was also observed in patients with acute lymphoblastic leukemia (HR 0.81; 95%CI 0.66–0.92, p: 0.045) [30]. Rahbar A et al. also described an inverse association between multiforme glioblastoma and CMV infection [31], and Couzi et al. described a reduction in cancer risk in kidney transplants linked to an increase in Vδ2negϒδT^17^. More recently, a potential protective effect of CMV reactivation on HCC and LT was described by Hsu et al. In that retrospective study, CMV reactivation, as measured by pp65 antigenemia, was associated with lower HCC recurrence after LT [14]. A significantly superior 5-year recurrence-free survival rate was observed in CMV antigenemia-positive patients compared to those who were negative (89% vs. 79%, p < 0.005). Our study shows that CMV reactivation is independently associated with reduced HCC recurrence, even after adjusting for other clinical and statistically significant factors. In addition, CMV reactivation also showed a trending protective effect on survival in association with other known factors such as AFP at LT and nodule size at explant. Hypothetically, CMVr could trigger a cross-reactive immunological response that might simultaneously reduce HCC recurrence. Patients who died due to HCC recurrence as opposed to those who died due to other causes, had lower rates of CMV reactivation (23% vs. 31.2%). Of note, cumulative exposure to tacrolimus was comparable in patients with and without CMVr thus eliminating the potential confounding effect of immunosuppression on the relationship between CMVr and HCC recurrence.

In our study, more than a third of the entire cohort suffered CMV reactivation or primary infection after a median of 41.5 days after LT, with need of antiviral treatment in 66% of the patients, and only 5.6% of the patients developed CMV disease. In fact, CMV DNA levels at first CMV reactivation were relatively low (median: 758(IQR: 405–2,340). Some studies have reported that low CMV levels without need for immediate treatment is protective by increasing the number and the activity of CMV-antigen-specific T cells [32], thereby hypothesizing a potential oncological protective effect by the above-described mechanisms without a deleterious effect on CMV control. In line with these results, in a recent *post hoc* analysis of a randomized controlled trial in D+/R-recipients that compared preemptive prophylaxis versus antiviral therapy, CMV DNAemia at six- and 12-months post-transplant were significantly higher in the group treated with universal prophylaxis as opposed to the preemptive approach and the higher DNAemia was also associated with increased mortality, suggesting a possible protective role for pre-emptive therapy secondary to an improved CMV-specific immunity while on preemptive versus prophylaxis [33]. Low-level CMV replication early after liver transplantation may enhance CMV-specific immunity, contribute to DNAemia control, and reduce inflammatory alloimmune responses and immunosenescence, which could ultimately impact survival, findings that are consistent with those observed in our study. Despite the fact that pre-emptive therapy is logistically more complex, practical real-world implementations have been recently advised [34]. Some studies have even postulated that universal prophylaxis could be harmful by delaying immune reconstitution against CMV [35]. However, facilitating CMV reactivation to diminish HCC recurrence may not be advisable until the underlying mechanisms are fully understood.

The use of immunotherapy in the LT arena when HCC recurs is still limited due to an enhanced risk of rejection [36, 37]. If the association between CMV reactivation and HCC recurrence is confirmed in larger, prospective multicenter studies, a potential use of oncolytic CMV therapies such as vaccine vectors, or a controlled preemptive approach could become a real strategy, at least for patients with a high risk of recurrence [37, 38].

As with other studies, additional known risk factors predicted HCC recurrence [1, 2, 5, 23, 39–41], including microvascular invasion and tumor size at explant. We also found that the use of DCD donors or the MELD score impacted HCC recurrence There is controversy regarding DCD and HCC recurrence [42–45]. A double ischemia impact, that could exacerbate liver tumor growth and favor metastasis through marked activation of cell adhesion, invasion, and angiogenesis pathways [42], could explain this association. However, we acknowledge caution is necessary when assessing this association given the small number of DCDs and a temporal bias (learning curve) [42–44]. Regarding the association between low MELD and lower HCC recurrence, it is possibly related to longer waiting time in this setting which provides an opportunity to better select patients with less aggressive tumor biology [46, 47].

Our study has some limitations. First, the retrospective approach and the multicenter participation have introduced heterogeneity related to CMV monitoring, diagnosis, including varying CMV detection methods and management. We made however a significant effort to ensure that the centers participating in this study followed a similar approach regarding CMV management, in accordance with international consensus [12]. Second, although HCC surveillance was done by each center practice, it is likely that it was not misdiagnosed due to the clinical relevance of HCC recurrence and close follow-up of the LT patients. In addition, information regarding immunosuppression including tacrolimus cumulative exposure was only available in a subgroup of patients and could not be controlled in the multivariable analysis.

In conclusion, CMVr reduces the risk of tumor recurrence in patients with HCC undergoing LT, particularly among patients showing other well-known risk factors such as increased tumor burden, microvascular invasion, or increased AFP at transplant. The most plausible mechanism involves immune-regulation pathways triggered by CMV although future studies are required to fully unravel the pathogenesis.

## Data Availability

The raw data supporting the conclusions of this article will be made available by the authors, without undue reservation.

## Supplementary Material

SUPPLEMENTARY FIGURE S1 Hepatocellular Carcinoma Free Survival.

SUPPLEMENTARY FIGURE S2 Survival according to hepatocellular carcinoma recurrence.

## Abbreviations

AFP, alpha fetoprotein; BMI, Body Mass Index; CMV, cytomegalovirus; CMVr, Cytomegalovirus reactivation after Liver transplantation; CI, confidence Interval; CNI, calcineurin inhibitors; CVE, Cardiovascular events; D, Donor; DCD, Donation after Circulatory Death; eGFR, estimated Glomerular Filtration Rate; HBV, Hepatitis B Virus; HCC, Hepatocellular carcinoma; HCV, Hepatitis C Virus; LT, liver transplantation; MASH, Metabolic dysfunction-associated steatohepatitis; MELD, Model for End-stage Liver Disease; mTORi, mammalian target of rapamycin inhibitors; R, Recipient; SD, Standard Deviation; VL, Viral load.
