# Supplementary material for: Cytomegalovirus Reactivation Is Associated With Lower Rates of Hepatocellular Carcinoma Recurrence After Liver Transplantation
Source: Transpl Int. 2025 Jun 10;38:14553. doi: 10.3389/ti.2025.14553 (PMC12185357; doi:10.3389/ti.2025.14553)
Supplement: Supplementary file 1 [file Table1.docx]

**Supplementary table 1:** **Factors associated with HCC recurrence (Cox simple,** **HR 95%, CI, p-value)**

| **Univariate anaysis** | | | |
| --- | --- | --- | --- |
|  | **HR** | **95%CI** | **P-value** |
| **Sex (men)** | 2.55 | 1.04-6.27 | **0.042*** |
| **Donor type (DCD)** | 2.33 | 0.95-5.75 | **0.065** |
| **HBV etiology** | 1.74 | 0.97-3.11 | **0.065** |
| **AFP at WL inclusion** | 1.000 | 1.001-1.002 | **0.008**** |
| **AFP at LT** | 1.000 | 1.001-1.002 | **0.002**** |
| **Milan”in” Criteria** | 0.41 | 0.26-0.66 | **<0.002***** |
| **Up to Seven criteria** | 0.4 | 0.16-0.98 | **0.044*** |
| **Retreat Score**  **4-8 points** | 3.9 | 2.4-6.4 | **<0.001***** |
| **Micro-vascular invasion**  **at explant** | 4.15 | 2.71-6.37 | **<0.001***** |
| **MELD score** | 0.95 | 0.91-0.99 | **0.020*** |
| **Number of nodules at last imaging** | 1.28 | 1.06 – 1.53 | **0.009**** |
| **Size of nodules at last imaging** | 1.02 | 1.01 – 1.04 | **0.005**** |
| **Number of nodules at explant** | 1.06 | 1.02 – 1.10 | **0.004**** |
| **Size of nodules at explant** | 1.04 | 1.03 – 1.06 | **<0.001***** |
| **Moderate or poor differentiation at explant** | 2.05 | 1.32-3.18 | **0.001**** |
| **Downstaging** | 2.47 | 1.58-3.85 | **<0.001***** |
| **CMV reactivation** | 0.65 | 0.4-1.05 | **0.077** |

*p<0.05; **p<0.01; ***p<0.001

AFP: alpha-fetoprotein; CMV: cytomegalovirus, HBV: Hepatitis B virus, LT: Liver transplantation, WL: Waiting list
