# Supplementary figures and images for: Cytomegalovirus Reactivation Is Associated With Lower Rates of Hepatocellular Carcinoma Recurrence After Liver Transplantation
Source: Transpl Int. 2025 Jun 10;38:14553. doi: 10.3389/ti.2025.14553 (PMC12185357; doi:10.3389/ti.2025.14553)

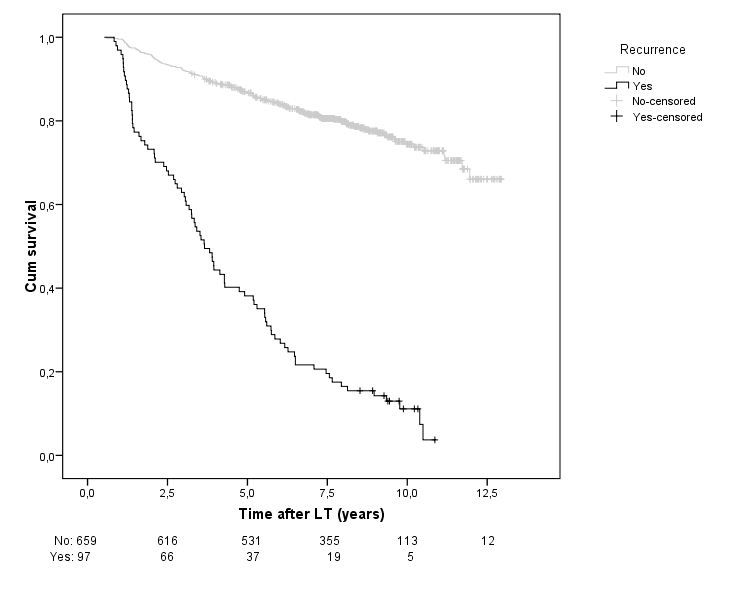

Supplement: Supplementary file 2 [file Image2.tif]

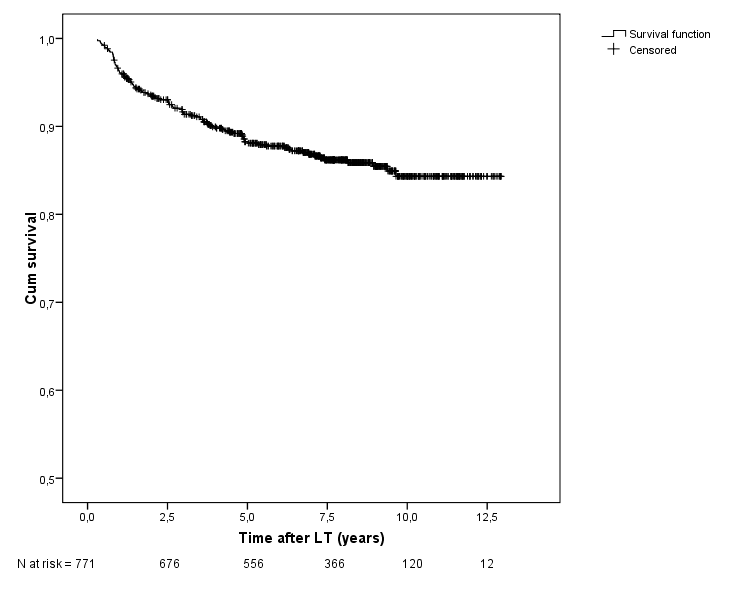

Supplement: Supplementary file 3 [file Image1.tif]
