# Supplementary material for: Cytomegalovirus Reactivation Is Associated With Lower Rates of Hepatocellular Carcinoma Recurrence After Liver Transplantation
Source: Transpl Int. 2025 Jun 10;38:14553. doi: 10.3389/ti.2025.14553 (PMC12185357; doi:10.3389/ti.2025.14553)
Supplement: Supplementary file 4 [file Table2.docx]

**Supplementary Table 2: Factors associated with survival. (Cox simple HR 95%, CI, p-value).**

|  | **Cox regression, hazard ratio (HR), 95%CI** | | |
| --- | --- | --- | --- |
|  | **HR** | **95% CI** | **p-value** |
| **Donor age (years)** | 1.011 | 1.003 – 1.020 | **0.006**** |
| **Recipient age (years)** | 1.018 | 0.998 – 1.039 | 0.076 |
| **Sex (men)** | 1.73 | 1.05 – 2.83 | **0.030*** |
| **DCD** | 1.68 | 0.83 – 3.40 | 0.151 |
| **Diabetes pre-LT** | 1.07 | 0.81 – 1.40 | 0.641 |
| **Other etiologies** | 1.10 | 0.85 – 1.44 | 0.469 |
| **Mash etiology** | 0.65 | 0.24 – 1.74 | 0.387 |
| **HCV etiology** | 0.78 | 0.60 – 1.01 | 0.056 |
| **HBV etiology** | 0.80 | 0.49 – 1.31 | 0.368 |
| **Alcohol etiology** | 1.63 | 1.25 – 2.14 | **<0.001***** |
| **Milan-In** | 0.69 | 0.48 – 0.98 | **0.038*** |
| **Up to Seven** | 0.53 | 0.27 – 1.04 | 0.063 |
| **Retreat score** |  |  |  |
| **0-3** | 1 |  |  |
| **4-8** | 2.17 | 1.55 – 3.04 | **<0.001***** |
| **eGFR (ml/min)** | 1.00 | 0.99 – 1.01 | 0.913 |
| **MELD score** | 0.99 | 0.97 – 1.02 | 0.900 |
| **AFP at WL** | 1.001 | 1.000 – 1.003 | **0.036*** |
| **AFP at LT** | 1.002 | 1.001 – 1.003 | **<0.001***** |
| **Mismatch CMV** |  |  |  |
| **1,2,3** | 1 |  |  |
| **4** | 0.96 | 0.61 – 1.53 | 0.876 |
| **Number of nodules at last imaging** | 1.14 | 1.01 – 1.28 | **0.045*** |
| **Nodule size at last imaging** | 1.01 | 0.99 – 1.02 | 0.160 |
| **Number of nodules at explant** | 1.02 | 0.98 – 1.07 | 0.286 |
| **Nodule size at explant** | 1.02 | 1.01 – 1.03 | **<0.001***** |
| **CMV prophylaxis** | 0.96 | 0.69 – 1.33 | 0.808 |
| **CMV primary infection** | 1.25 | 0.75 – 2.08 | 0.385 |
| **CMV reactivation** | 0.78 | 0.58 – 1.05 | 0.096 |
| **Primo and reactivation** | 0.83 | 0.63 – 1.11 | 0.210 |
| **Micro-vascular invasion** | 2.00 | 1.44 – 2.79 | **<0.001***** |
| **Differentiation grade** |  |  |  |
| **-Good or total tumor necrosis** | 1 |  |  |
| **-Moderate or poor** | 1.46 | 1.11 – 1.92 | **0.007**** |
| **Bridging** |  |  |  |
| **-No treatment** | 1 |  |  |
| **-Other treatments for bridging** | 0.99 | 0.76 – 1.31 | 0.974 |
| **Downstaging** | 1.55 | 1.11 – 2.18 | **0.011*** |
| **CMV disease** | 1.39 | 0.71 – 2.72 | 0.341 |

*p<0.05; **p<0.01; ***p<0.001

AFP: Alpha-fetoprotein; CMV: cytomegalovirus, DCD: donation after circulatory death, HBV: Hepatitis B virus, LT: Liver transplantation, WL: Waitlisting
