# Supplementary material for: Cytomegalovirus Reactivation Is Associated With Lower Rates of Hepatocellular Carcinoma Recurrence After Liver Transplantation
Source: Transpl Int. 2025 Jun 10;38:14553. doi: 10.3389/ti.2025.14553 (PMC12185357; doi:10.3389/ti.2025.14553)
Supplement: Supplementary file 5 [file Table3.docx]

**Supplementary Table 3:**

**HCC recurrence based on need of antiviral therapy in those with CMV reactivation:**

|  | HCC recurrence | HCC-Recurrence free survival | | | |
| --- | --- | --- | --- | --- | --- |
|  |  | 1 year | 3 years | 5 years | 7 years |
| CMVr and no treatment* | 10% (=6/60) | 96.7% | 93.2% | 89,6% | 89.6% |
| CMVr and treatment** | 9.5% (=15/158) | 96.8% | 93.5% | 91.3% | 90.4% |

*Patients developing CMVr with spontaneous clearance not requiring antiviral therapy.

**Patients developing CMVr followed by antiviral therapy
